# Shaped Singular Spectrum Analysis for Quantifying Gene Expression, with Application to the Early *Drosophila* Embryo

**DOI:** 10.1155/2015/689745

**Published:** 2015-03-19

**Authors:** Alex Shlemov, Nina Golyandina, David Holloway, Alexander Spirov

**Affiliations:** ^1^Faculty of Mathematics and Mechanics, St. Petersburg State University, Universitetsky Pr. 28, Peterhof, St. Petersburg 198504, Russia; ^2^Mathematics Department, British Columbia Institute of Technology, 3700 Willingdon Avenue, Burnaby, BC, Canada V5G 3H2; ^3^Computer Science and CEWIT, SUNY Stony Brook, 1500 Stony Brook Road, Stony Brook, NY 11794, USA; ^4^The Sechenov Institute of Evolutionary Physiology & Biochemistry, Torez Pr. 44, St. Petersburg 194223, Russia

## Abstract

In recent years, with the development of automated microscopy technologies, the volume and complexity of image data on gene expression have increased tremendously. The only way to analyze quantitatively and comprehensively such biological data is by developing and applying new sophisticated mathematical approaches. Here, we present extensions of 2D singular spectrum analysis (2D-SSA) for application to 2D and 3D datasets of embryo images. These extensions, circular and shaped 2D-SSA, are applied to gene expression in the nuclear layer just under the surface of the *Drosophila* (fruit fly) embryo. We consider the commonly used cylindrical projection of the ellipsoidal *Drosophila* embryo. We demonstrate how circular and shaped versions of 2D-SSA help to decompose expression data into identifiable components (such as trend and noise), as well as separating signals from different genes. Detection and improvement of under- and overcorrection in multichannel imaging is addressed, as well as the extraction and analysis of 3D features in 3D gene expression patterns.

## 1. Introduction

While the availability of genome sequences has drastically revolutionized biological and biomedical research, our understanding of how genes encode regulatory mechanisms is still limited. Embryonic development depends critically on such regulatory mechanisms in order for cells to differentiate in the correct positions and at the correct times. Global understanding of gene regulation in development requires determining at cellular resolution in vivo when and where each gene is expressed. New dynamic, cellular resolution atlases will address the question of how gene transcription factors influence expression patterning [1].

With the development of automated microscopy technologies in recent years the volume and complexity of image data have increased to the level that it is no longer feasible to extract information without using computational tools. Biologists increasingly rely on computer scientists to come up with new solutions and software [2]. Such computational tools have been essential for processing the images generated by high-throughput microscopy of large numbers and varieties of biological samples under a variety of conditions. Recent advances in labeling, imaging, and computational image analysis are allowing quantitative measurements to be made more readily and in much greater detail in a range of organisms (e.g.,* Arabidopsis*,* Ciona*,* Drosophila*,* C. elegans*, mice,* Platynereis*, and zebrafish) [1, 3–6]. In particular, imaging of single intact small organisms, like* Drosophila* and* C. elegans*, is now feasible with high resolution in two dimensions, three dimensions, and across time, resulting in massive image data sets available for comprehensive computational analysis.

These large-scale quantitative data sets provide new insights to address many fundamental questions in developmental biology. The initial inputs for deriving quantitative information of gene expression and embryonic morphology are usually raw image data of stained fluorescent markers in fixed material. These raw image sets are then analyzed by computational algorithms that extract features such as cell location, cell shape, and gene product concentration. Ultimately, the most powerful way to analyze 3D spatial data in biology is by developing and applying new sophisticated mathematical approaches, allowing for the rigorous comparison of multiple quantitative features [8, 9].

In this publication, we introduce new computational tools to analyze gene patterning for three spatial dimension datasets, applied to early* Drosophila* embryos. These tools are an extension of two-dimensional singular spectrum analysis (2D-SSA).

*Introduction to the Method*. Singular spectrum analysis [10–15] was originally suggested as a method for decomposition of time series into a sum of identifiable components such as trend (or pattern), oscillations, and noise. One advantage of this method is that it does not need a noise model to be given a priori. We decompose the data series into a set of elementary series, analyze them, choose appropriate components, and finally sum the identifiable components together in classes. As an example, selection of smooth components can produce adaptive smoothing. SSA is very useful for exploratory analysis since the method can deal with modulated noise, that is, noise that can depend on trend values (e.g., has a multiplicative nature).

Recently SSA was extended for analysis of two-dimensional objects (2D-SSA), for example, digital images [16, 17]. Decomposition of images is more complicated compared to time series analysis due to variability of 2D patterns. But methods which are easily controlled and adaptive, such as 2D-SSA, can have broad applicability.

2D-SSA has much in common with the 2D-ESPRIT method (see [18]), which is based on the parametric form of images and has many applications. 2D-SSA and related subspace-based methods are applied in texture analysis [19], seismology [20], spatial gene expression data [21], and medical imaging [22].

The paper [23] applied 2D-SSA to the analysis of digital terrains in geology and demonstrated that 2D-SSA is a useful tool for analyzing different levels of details in surface data. Later, based on the theory given in [17], 2D-SSA was applied to gene expression data to separate nuclear noise from expression trend [21].

The papers [24, 25] present extensions of 2D-SSA which increase the range of SSA applications. In the present paper, we demonstrate how these extensions can be applied to analyzing gene expression data.

This paper is structured as follows.  Section 2 describes the data sets which were analyzed.  Section 3 describes the new methodology, and Sections 4 and 5 demonstrate the approach on several examples.

The new approaches described here, circular and shaped 2D-SSA, are particulary applicable to cylindrical surfaces (as used for* Drosophila* embryos), to avoid edge effects and patterns of irregular shape. For example, the area of good quality data in an image (e.g., without oversaturation) can be nonrectangular and even have gaps. Also, since the planar projection of a* Drosophila* embryo is nearly elliptical, the ability to analyze nonrectangular shapes can be useful.

Section 4 deals with the problem of detection and improvement of under- and overcorrection in multichannel imaging, while Section 5 considers the problem of analysis of stripe shapes for the even skipped gene.  Section 6 contains a short discussion and conclusions.

## 2. Materials

Data are taken from the Berkeley Drosophila Transcription Network Project (BDTNP) [4], which contains three-dimensional (3D) measurements of relative mRNA concentration for 95 genes in early development (including* snail* (*sna*)) and the protein expression patterns for four genes (bicoid, giant, hunchback (*hb*), and Krüppel (*Kr*)) during nuclear cleavage cycles 13 (C13) and 14 (C14A). BDTNP Release 2 contains individual datasets (PointCloud files) for 2830 embryos (http://bdtnp.lbl.gov/Fly-Net/bioimaging.jsp). These data were registered to the coordinates of 6078 nuclei on the embryo cortex and presented as an integrated dataset (VirtualEmbryo file, with tools for visualization and analysis). Embryos were fixed and fluorescently stained to label the mRNA expression patterns of two genes plus nuclear DNA. One of the genes stained was either even skipped (*eve*) or* fushi tarazu* (*ftz*), which were used as fiduciary markers for subsequent spatial registration.

## 3. Methods

### 3.1. 2D Singular Spectrum Analysis

We will follow the common structure of 2D-SSA algorithms described in [24, 25]. This common structure consists of embedding, decomposition, grouping, and reconstruction steps. Input for a 2D-SSA algorithm consists of an image *𝕏* and the shape of a moving window (which is the main algorithm parameter). The output of a 2D-SSA algorithm is the decomposition of *𝕏* into identifiable components of the form *𝕏* = *𝕏*_1_ + ⋯+*𝕏*_*s*_.

*Common Scheme of SSA-Like Algorithms*

(*1) Embedding Step*. Construction of the trajectory matrix **X** = *𝒯*(*𝕏*) ∈ *H*, where *H* is a space of structured Hankel-like matrices. The structure of the matrix **X** (and the space *H*) depends on the algorithm modification and on the moving window. Generally speaking, the columns of the trajectory matrix consist of the windows moving along the image, transformed to vectors by a fixed order of window elements. In a sense, the window size reflects the resolution of the method; that is, larger windows lead to more detailed decompositions. 

(*2) Decomposition Step*. Singular value decomposition (SVD) of the trajectory matrix $X=\sum_{i=1}^{d}\sqrt{\lambda_{i}}U_{i}V_{i}^{⊤}=\sum_{i=1}^{d}X_{i}$. Here $\left(\sqrt{\lambda_{i}},U_{i},V_{i}\right)$ are so-called eigentriples (abbreviated as ET) and consist of singular values, left and right singular vectors of **X**. The eigenvectors can be transformed back to the window form. This means that we can consider eigenvectors as images and call them eigenimages. 

(*3) Grouping Step*. Partition {1,…, *d*} = ∐_*j*=1_^*s*^ *I*_*j*_ and grouping of summands in the SVD decomposition to obtain a grouped matrix decomposition **X** = ∑_*j*=1_^*s*^ **X**_*I*_*j*__, where **X**_*I*_ = ∑_*k*∈*I*_ **X**_*k*_. The grouping with *I*_*j*_ = {*j*} is called elementary. The aim of this step is to group the SVD components to obtain an interpretable decomposition of the initial object. This can be performed by means of analysis of eigentriples. 

(*4) Reconstruction Step*. Decomposition of the initial image *𝕏* = *𝕏*_1_ + ⋯+*𝕏*_*s*_, where *𝕏*_*j*_ = *𝒯* ^−1^ *ℋ*(**X**_*I*_*j*__); *ℋ* is the operator of projection on the space *H* (e.g., hankelization in the 1D case); *ℋ*(**X**_*I*_) = ∑_*i*∈*I*_ *ℋ*(**X**_*i*_) holds.

Let us explain the sense of the embedding operator *𝒯* for the 1D case, since it is simpler and demonstrates the general methodology. For a one-dimensional series *𝕏* = (*x*_1_,…, *x*_*N*_), we take moving 1D windows of length *L* and construct the columns of the trajectory matrix in the forms *X*_1_ = (*x*_1_,…,*x*_*L*_)^T^, *X*_2_ = (*x*_2_,…,*x*_*L*+1_)^T^, and so on. From these *K* = *N* − *L* + 1 lagged vectors we gather a Hankel matrix with equal numbers on antidiagonals called the trajectory matrix

(1) $$\begin{array}{c} T_{\text{SSA}}\left(X\right)=\left(\begin{array}{ccccc} x_{1} & x_{2} & x_{3} & \cdots & x_{K}\\ x_{2} & x_{3} & x_{4} & \cdots & x_{K+1}\\ x_{3} & x_{4} & x_{5} & \cdots & x_{K+2}\\ \vdots & \vdots & \vdots & \ddots & \vdots \\ x_{L} & x_{L+1} & x_{L+2} & \cdots & x_{N} \end{array}\right). \end{array}$$

It is well known that Hankel matrices are related to series which consist of sums of products of polynomials, exponentials, and sine waves and the problem is to separate this sum into addends. If we can separate exponential and polynomial approximations from the residual, then we can extract trends and patterns. If we are able to separate sine waves with different frequencies, then we can construct a decomposition on components with different frequency ranges.

The singular value decomposition (SVD) of the trajectory matrix constructs a sequence of elementary matrices, which provides the best approximations of the initial matrix and, in a sense, of the initial series: **X**_1_, **X**_1_ + **X**_2_, and so on. Thus, we obtain the optimal decomposition, which is adaptive to the initial series. Note that the maximal number of the decomposition elements is equal to min⁡(*L*, *K*). SSA theory explains why we can group the elementary components in the SVD expansion to solve such problems as, for example, smooth approximation and extraction of regular oscillations.

After a proper grouping, we obtain a matrix **X**_*I*_, which is close to a Hankel matrix, but not exactly Hankel. We can find the Hankel matrix closest to **X**_*I*_ = {*y*_*ij*_} by hankelization, that is, by averaging values by antidiagonals. Thus, we obtain the series consisting of *y*_11_, (*y*_12_ + *y*_21_)/2, (*y*_13_ + *y*_22_ + *y*_31_)/3, and so on. The* m*th term is determined as ∑_*i*,*j*∈*𝒜*_*m*__ *y*_*ij*_/|*𝒜*_*m*_|, where *𝒜*_*m*_ = {*i*, *j* : 1 ≤ *i* ≤ *L*, 1 ≤ *j* ≤ *K*, *i* + *j* = *m* + 1}.

The role of *L* is as follows. Small *L* provides a decomposition to a small number of components, which mostly differ by frequency, and where the leading components present slowly varying series like the trend. Larger *L* leads to more detailed decomposition. This gives more chance to extract a component; however, some components can mix. Therefore, if the data series has a trend with a complex form or has periodicities with complex modulation, then window lengths should be moderate.

These generalities also hold for the case of 2D-SSA. In practice, the difference between 1D and 2D is in the construction of the trajectory matrices, which are quasi-Hankel, in particular Hankel-block-Hankel. The moving window is two-dimensional, for example, a rectangle. In this paper, we introduce circular SSA, for treating rectangles with periodic boundary conditions, for example, data sets on cylindrical geometries. Small window size corresponds to smoothing. We can take into consideration the structure of the image in different directions by choosing different sizes in different directions. The trajectory matrix is constructed from vectorized windows of arbitrary shape moving within the whole image (including circular domains, for periodic boundary conditions).

### 3.2. Particular Cases

For a rectangular image, with a rectangular window which moves within the image boundaries, we obtain the standard 2D-SSA method. If the image and the window are of arbitrary shape, the shaped version of 2D-SSA is applied [25]. If the window can cross the boundary of the image, we obtain a circular version of 2D-SSA.

For example, let us take an image (a matrix in the mathematical sense)

(2) $$\begin{array}{c} X=\left(\begin{array}{ccc} 1 & 2 & 3\\ 4 & 5 & 6\\ 7 & 8 & 9 \end{array}\right) \end{array}$$

and the window of size 2 × 2. Then we have a set of 4 windows in the ordinary version, $\left(\begin{array}{cc} 1 & 2\\ 4 & 5 \end{array}\right)$, $\left(\begin{array}{cc} 2 & 3\\ 5 & 6 \end{array}\right)$, $\left(\begin{array}{cc} 4 & 5\\ 7 & 8 \end{array}\right)$, and $\left(\begin{array}{cc} 5 & 6\\ 8 & 9 \end{array}\right)$, and two additional windows, $\left(\begin{array}{cc} 7 & 8\\ 1 & 2 \end{array}\right)$, $\left(\begin{array}{cc} 8 & 9\\ 2 & 3 \end{array}\right)$, in the circular case. For the circular case, the trajectory matrix will have the form

(3) $$\begin{array}{c} X=\left(\begin{array}{cccccc} 1 & 2 & 4 & 5 & 7 & 8\\ 2 & 3 & 5 & 6 & 8 & 9\\ 4 & 5 & 7 & 8 & 1 & 2\\ 5 & 6 & 8 & 9 & 2 & 3 \end{array}\right). \end{array}$$

One can see that the 2D trajectory matrix consists of trajectory matrices from each matrix's row.

### 3.3. Choice of Parameters, Separability, and Component Identification

Approach to the choice of window size for one-dimensional time series is thoroughly described in [13, 26]. Recommendations for 2D objects are more complicated. For extraction of so-called objects of finite rank (sums of products of polynomials, exponentials, and sinusoids), which satisfy linear recurrence relations (LRRs), windows should be large, up to half of the object size. However, real-world patterns usually have complex form and satisfy LRRs only approximately and locally. The window needs to agree with this local character. In particular, sine waves are exactly governed by an LRR. However, if a 2D-sine wave has a slowly changing location, then only its local parts satisfy an LRR. The window sizes need to be in accordance with the scale of this locality. Choice of window size is always a balance between the local and the global scales of the data.

Generally, SSA can separate smooth patterns from noise for a wide variety of patterns. For regular patterns, 2D-SSA can be applied whether the pattern varies smoothly or sharply. However, if the pattern is not regular, variation needs to be smooth in order to use 2D-SSA for signal separation. Irregular pattern with sharp variation is poorly separated by 2D-SSA. If, however, the sharp change occurs in narrow area, this can be cut out, and the remaining data analyzed by shaped SSA, which is a version of 2D-SSA with a nonrectangular shape of the image or the window.

Elementary components are grouped based on their similarity to the data components being extracted. For regular components like sine waves, the number of elementary components can be calculated from theory. Also, patterns usually have a limited frequency range (usually lacking high frequencies). In general, therefore, leading elementary components with the appropriate frequency characteristics are ascribed to pattern.

In this paper we show how 2D-SSA can be used to remove noise, to separate regular oscillations from slowly varying patterns (for correcting erroneous unmixing procedures), and to extract stripes for their further analysis. Shaped SSA allows for the analysis of complex patterns by splitting images into several parts.

*Drosophila* early gene expression (before the midblastula transition) produces smooth and simple patterns suitable for 2D-SSA processing. A number of web resources have such datasets (BDTNP BID [4], Fly-FISH http://fly-fish.ccbr.utoronto.ca [27], FlyEx http://urchin.spbcas.ru/flyex [28]; see also [29, 30]). Shaped SSA can also be useful for a common subset of this data, in which patterns fall sharply to zero. In these cases, subregions can be excised or analyzed separately from the whole image. The gene* sna* is a typical* Drosophila* example seen in the BDTNP BID; such compact patterns are also seen in other experimental organisms, such as the nine zebrafish genes [31]. We expect 2D-SSA and shaped SSA to therefore have broad applicability to image processing in developmental biology.

The problem of unmixing expression patterns from two different genes in one image [32] requires additional conditions. Specifically, information is needed on the unmixed expression of each gene (i.e., data from one gene in the absence of the other gene). If the two genes have slowly varying patterns, they cannot readily be separated by SSA. In such cases, SSA cannot be used to detect or correct errors in mixed images. However, SSA is an effective unmixing method for cases in which one gene has an approximately regular structure, and this differs from the structure of the other gene. In this paper, we apply SSA to signal unmixing and image correction for such cases from* Drosophila* data.

### 3.4. Data Preprocessing

Initially, the data for 2D-SSA analysis should be measured on a regular grid. Data for gene expression are measured at nuclei, which are not regularly located on a 3D surface of embryo (which is roughly ellipsoidal in shape). The first step of preprocessing is a cylindrical projection of the data (centred on the major axis of the ellipsoid; the major axis of the embryo is found by principal component analysis). We then interpolate the data to a regular grid on this cylinder. We analyze a central region of the cylinder, in order to avoid corruptions near the poles from the ellipsoid to cylinder transformation. After 2D-SSA decomposition, we interpolated the data back onto the nuclear centers. This interpolation is performed for smooth components; residuals are calculated as the difference between the initial data and interpolated smooth components.

Interpolation involves Delaunay triangulation followed by linear interpolation of nuclear centers to the triangulation.

### 3.5. Implementation

The algorithms are implemented in the Rssa and BioSSA packages in* R*. Rssa is a general-purpose package containing effective implementation of singular spectrum analysis and its 2D extensions. 2D-SSA algorithms are time- and memory-consuming and therefore it is very important to have an effective implementation. A description of Rssa with examples can be found in [24, 33]. The* R*-package BioSSA is an addition to Rssa for application to fly embryo gene expressions data and is briefly described at http://biossa.github.io/.

## 4. Periodic Patterns Produced by Unmixing Algorithms

Different emission spectra for fluorescent probes allows for the simultaneous staining for 3-4 gene products in embryonic tissues. Quantitative imaging projects [4, 30] use the same gene in one of these channels in all embryos, for reliable quantitative comparisons, registration, and so forth. The gene used for this marking in* Drosophila* embryos is commonly one of the pair-rule genes (such as* eve* or* ftz*), which have a characteristic periodic 7-stripe expression pattern.

Multichannel imaging suffers from an inherent problem of overlapping emission spectra (when the fluorescent markers are simultaneously excited (e.g., [34])), where light from more than one fluorescent dye is collected by a given acquisition channel. To computationally reduce this “crosstalk,” an automated channel unmixing method was developed and applied to the BDTNP data [32].

**Figure 1:**
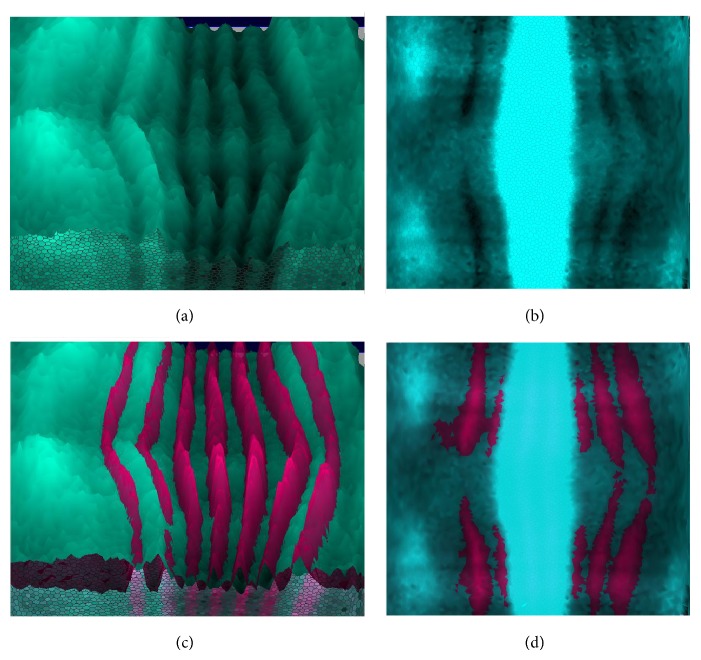
An example of overcorrection in gene expression data causing the subtraction of the reference gene pattern (the seven-striped* ftz* and* eve* patterns; dark magenta) from the pattern under study (*hb* and* Kr* gene products (transcription factors); light blue). Visualization by PointCloudXplore tools [7], BDTNP embryos* hb* “v5-s11512-2oc06-25” ((a) and (c)),* Kr* “v5-s12169-24oc07-22” ((b) and (d)); (c) is the same as (a) with added* ftz*; (d) is the same as (b) with added* eve*.

The problem with this approach in large scale projects with automatic data processing is that the unmixing parameters can end up being too high or too low. If the parameters are overestimated, unmixing produces an overcorrection, which is manifest as a partial subtraction of the common, reference pattern from the pattern of the second gene (the gene under study for the embryo). With periodic reference patterns (*eve*,* ftz*), this produces periodic grooves in the “unmixed” pattern.  Figure 1 shows the effects of such overcorrection in one of the BDTNP embryos.

**Figure 2:**
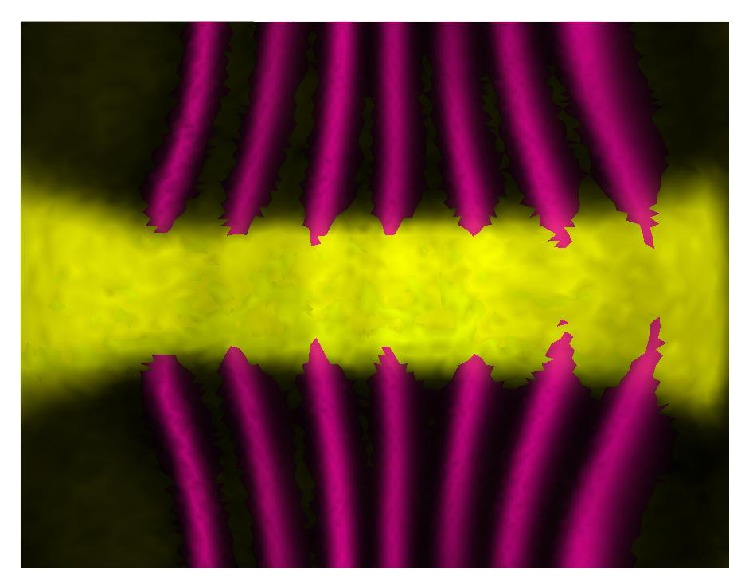
An example of undercorrection, in which the periodic reference gene pattern (*eve*; dark magenta) adds periodicity to the nonperiodic pattern under study (*sna* gene product; yellow). Visualization by PointCloudXplore. Embryo “v5-s10531-28fe05-07.”

On the other hand, if the unmixing parameters are underestimated, unmixing produces an undercorrection, which can be seen as an addition of the common, reference pattern to the pattern of the second gene (that one being studied in the given embryo).  Figure 2 shows an example of undercorrection on a BDTNP embryo.

Misestimation of the unmixing parameters can be seen to introduce periodicity in a number of BDTNP embryos from the 7-stripe* eve* or* ftz* reference patterns. The effect is strong enough to be seen in some images integrated from multiple embryos (such as Figure 2).

We now show how decomposition by circular 2D-SSA can be used to estimate and eliminate the periodic components caused by under- or overcorrection, using the examples of the BDTNP images in Figures 1 and 2.

### 4.1. Circular 2D-SSA,* hb* Corrupted by* ftz*, and Strong Overcorrection

**Figure 3:**
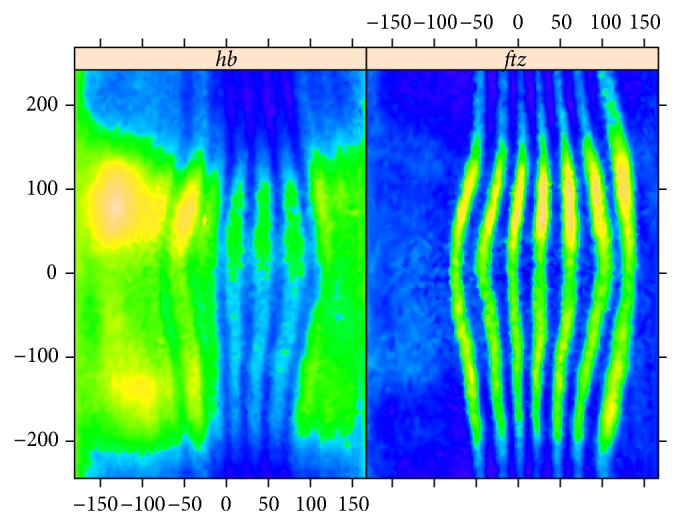
*hb* and* ftz*: original images of the “unrolled” cylindrical surface; the top values are a direct continuation of bottom values.

Figure 3 shows the original images for* hb* and* ftz* expressions from a BDTNP embryo (ID “v5-s11512-2oc06-25”). The natural* hb* trend is of low frequency; the natural pattern of* ftz* is of high frequency; crosstalk, with overcorrection in the unmixing algorithm, “bleeds” the high frequency* ftz* pattern into the* hb* pattern. These images are “unrolled” from the cylindrical projection of the data; therefore, the top and bottom edges connect (periodic boundary conditions).

We preprocess the images by interpolating to a regular grid (step 0.5%) and removing 20% from the left and 5% from the right (to focus on the stripe region). Use of circular 2D-SSA allows us to analyze the cylindrical dataset. We use a rectangular window of 25 × 10. In consideration of the regular oscillations along the anteroposterior (AP, horizontal) coordinate, the first window dimension, 25, is larger than the second dimension, 10.

**Figure 4:**
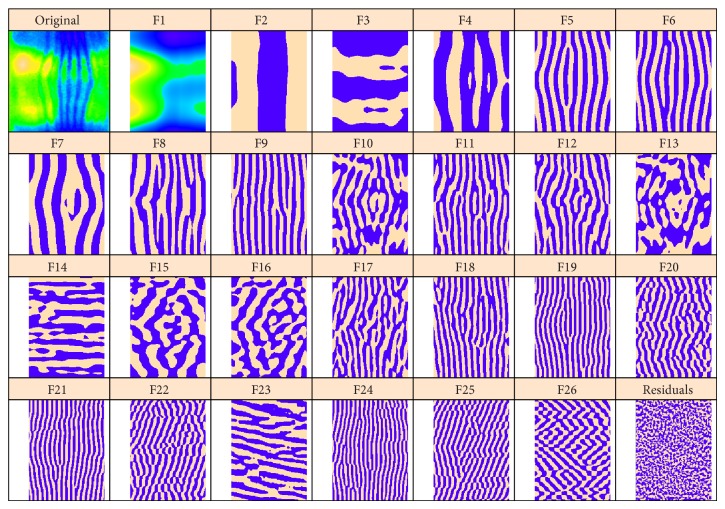
*hb*: the original image and the elementary components extracted by circular 2D-SSA. The original image and the leading component (F1) are colour-mapped according to the min and max expression levels. For more contrast, the remaining components are depicted in a binary format, with positive values in beige and negative values in purple.

**Figure 5:**
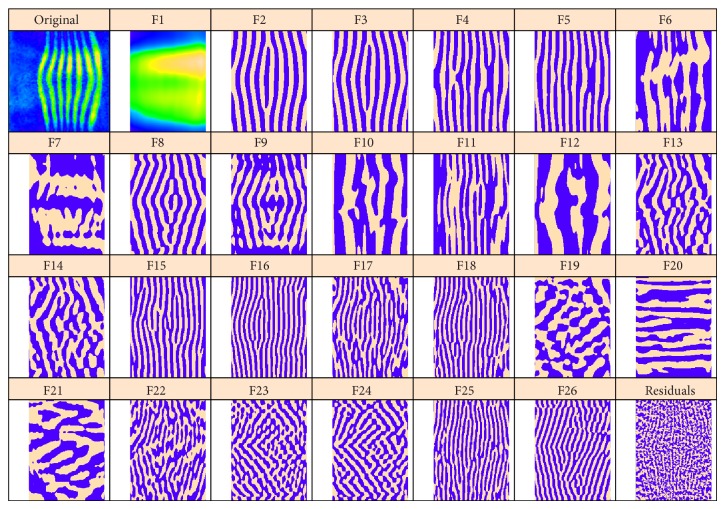
*ftz*: original image, F1 with the background; the remaining elementary components are depicted in a binary format.

Figure 4 presents 2D-SSA decomposition into elementary image components for* hb*; Figure 5 shows this for* ftz* (we depict the 26 largest components; the smaller components were not found to be significant in image reconstruction). Figure 4 contains a number of components with vertical stripes caused by or influenced by the* ftz* channel. If one compares elementary components of the* ftz* decomposition (Figure 5, striped components 2–5, 9–11, and 15–17) with the* hb* decomposition (Figure 4), it appears that* hb* components 1–4 are likely due to expression pattern, while components 5–9, 11, and probably 10, 12 are due to* ftz*-correction.

**Figure 6:**
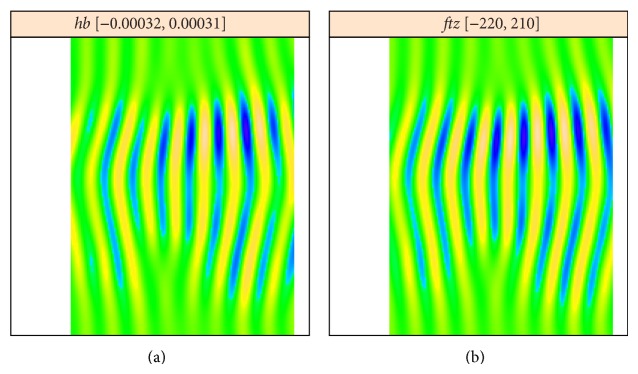
*hb* (a) and* ftz* (b): reconstruction from the main striped components 5 and 6 for the* hb* analysis, 2 and 3 for the* ftz* analysis. The stripes are out of phase for* hb* and* ftz*.

**Figure 7:**
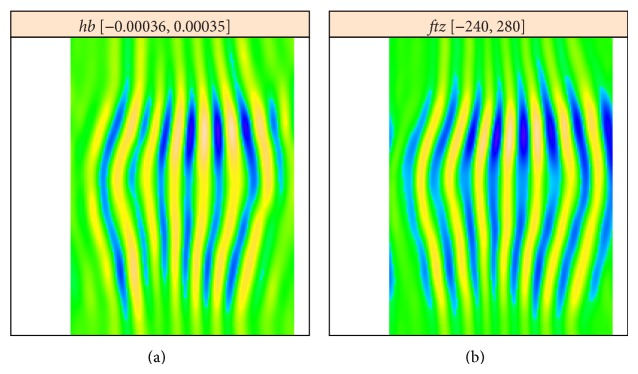
*hb* (a) and* ftz* (b): reconstruction from all striped components.

Figure 6 shows reconstructions from the leading high frequency components for each image, components 5 and 6 from Figure 4, components 2 and 3 from Figure 5. The reconstructions are very similar, but have opposite phases, indicating that the* hb* data was overcorrected.  Figure 7 is reconstructed from all striped components for each image; again, the patterns are very similar but of opposite phase.

**Figure 8:**
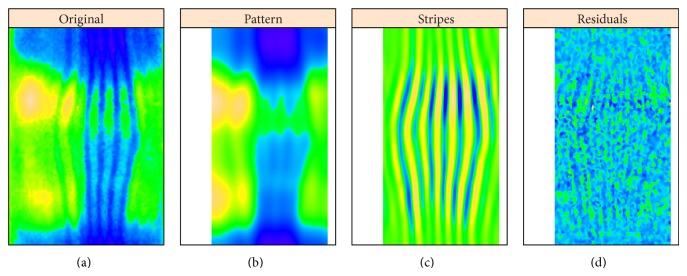
*hb* ((a) to (d)): original image, unstriped pattern, stripes, and residual noise.

Simultaneously, with removing stripes, this process also decomposes an image into pattern and noise (residuals): Figure 8 shows reconstruction of* hb* expression from the “unstriped” components 1–4, alongside the striped components (strongly affected by* ftz*) 5–12 and the residuals. Circular 2D-SSA provides a method for removing under- or overcorrection in the unmixing algorithm and therefore of clearing gene patterns from crosstalk effects. For an image without stripes, 2D-SSA produces a direct decomposition into pattern and noise. We show here that SSA decomposition is robust for data with crosstalk stripes.

### 4.2. Circular 2D-SSA,* Kr* Corrupted by* eve*, and Weak Overcorrection

**Figure 9:**
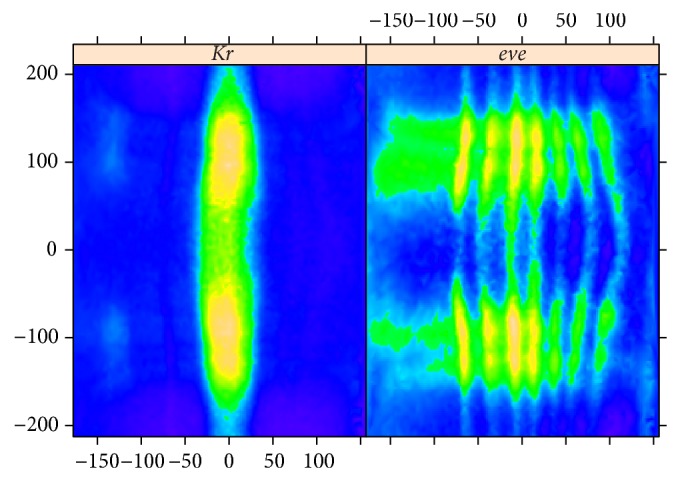
*Kr* and* eve*: original images.

In some cases, crosstalk stripes from the pair-rule reference marker are barely visible in the gene of interest. In these cases, circular 2D-SSA is still effective at removing artefacts from misestimation of the unmixing parameters.  Figure 9 shows images from an embryo “v5-s11512-2oc06-25” stained for* Kr* (gene of interest) mRNA and* eve* (reference marker) mRNA. In this case, there is weak overcorrection, with* eve* adding to apparent intensity in the* Kr* image.* Kr*, like* hb* (Figure 3), is a gap gene, with low frequency expression pattern, compared to the high frequency* eve* pair-rule pattern.

**Figure 10:**
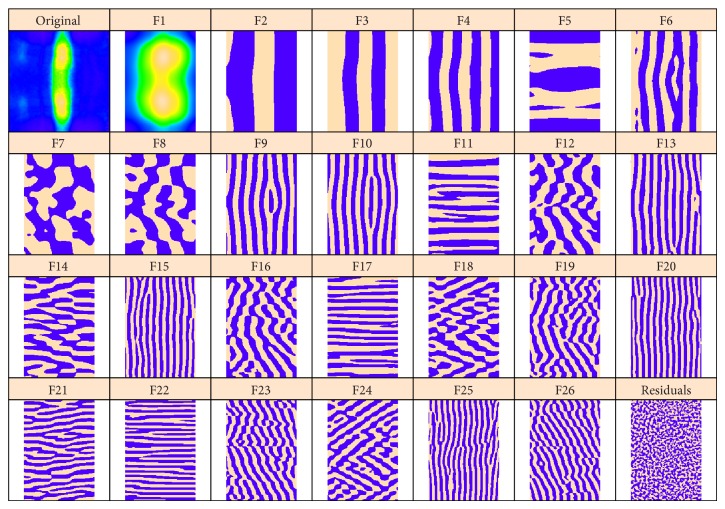
*Kr* gene expression, with circular 2D-SSA decomposition: original image and elementary components. As with Figures 11 and 10, the original image and leading component (F1) are colour-mapped according to min and max expression levels. For more contrast, the remaining components are depicted purple and beige.

**Figure 11:**
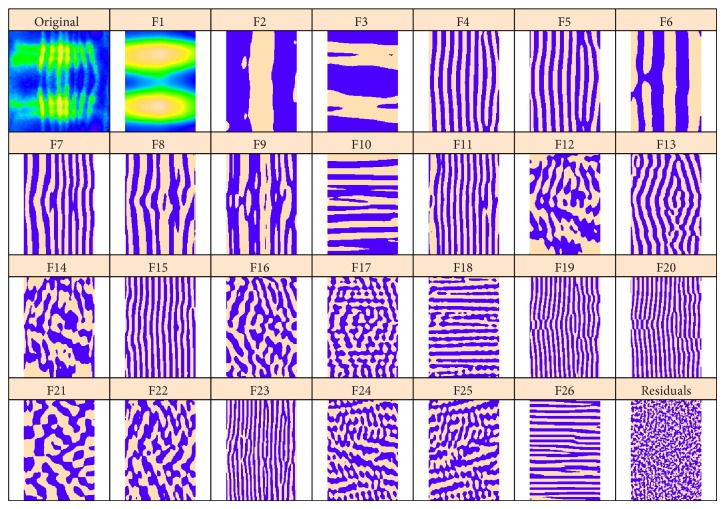
*eve* gene expression, with circular 2D-SSA decomposition: original image and elementary components.

We perform the same preprocessing and choose the same method parameters as in Section 4.1.  Figure 10 shows circular 2D-SSA (top and bottom edges are contiguous) decomposition into elementary components for* Kr*; Figure 11 shows this for the* eve* image.

**Figure 12:**
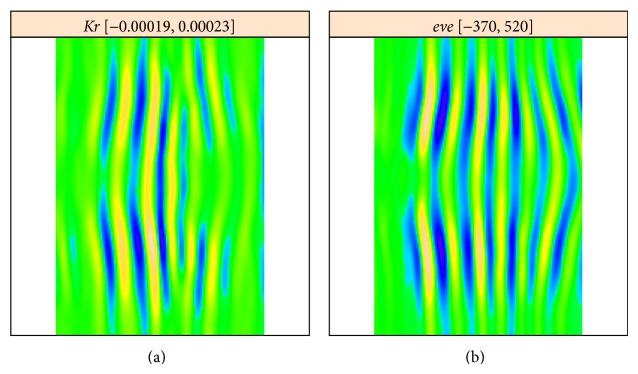
*Kr* and* eve*: reconstruction with stripe components, from the* Kr* image (a) and from the* eve* image (b). The frequencies correspond, but are out-of-phase, indicating overcorrection in the unmixing algorithm.

The decomposition in Figure 10 shows components with low frequency vertical stripes corresponding to the* Kr* signal, as well as high frequency stripes corresponding to* eve*. These high frequency stripes can be seen in the* eve* decomposition (Figure 11), in particular components 4-5, 7–9, 11, 13, 15, 19, and 20. Conversely,* Kr* crosstalk on the eve image is apparent in Figure 11 in components 9, 10, 13, 15, 20, and 25.  Figure 12 shows reconstructions using the stripe components from the images. Again, being a characteristic of overcorrection in the unmixing algorithm, these patterns are of comparable frequency, but of opposite phase.

**Figure 13:**
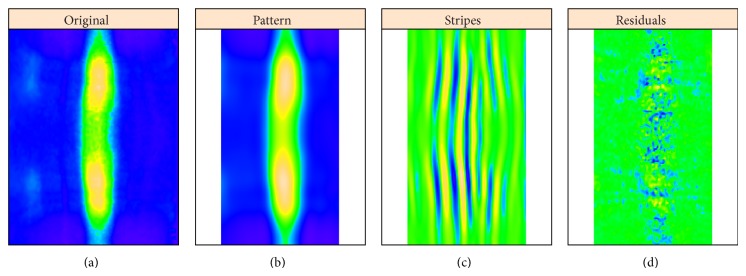
*Kr*: processing of the* Kr* expression image by circular 2D-SSA. ((a) to (d)): original image, pattern components (numbers 1–8), stripes (components 9, 10, 13, 15, 20, 25), and residual noise.

Figure 13 shows reconstruction of the* Kr* expression pattern from the circular 2D-SSA components. In the analysis of Figure 3, crosstalk overcorrection was strong and evident by eye. In Figure 9, the crosstalk stripes are not as evident by eye, but circular 2D-SSA is still effective for separating signal from the gene of interest (*Kr*) from the striped reference marker. Separation of pattern components leaves residual noise, for studying stochastic effects in gene expression.

### 4.3. Shaped 2D-SSA,* sna* Corrupted by* eve*, and Undercorrection

**Figure 14:**
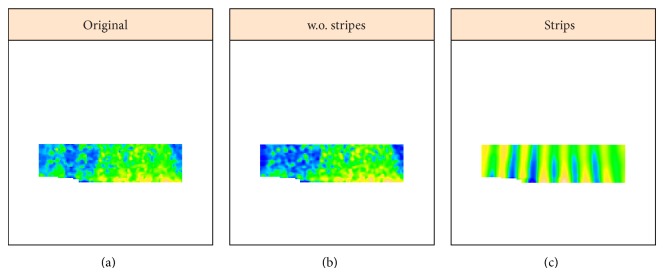
*sna* image, area 1, strong expression zone. ((a) to (c)): original image, reconstruction without stripes, and stripe components from the eve marker.

**Figure 15:**
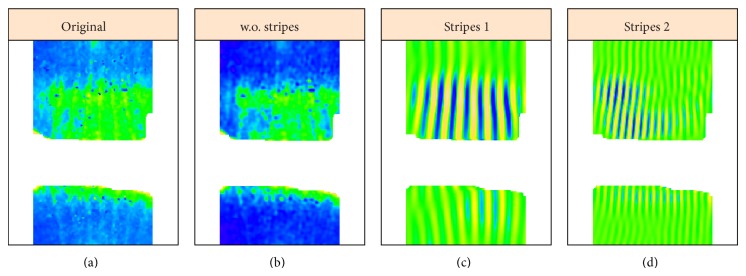
*sna* image, area 2, weak expression zone. ((a) to (d)): original image, reconstruction without stripes, and stripe components.

**Figure 16:**
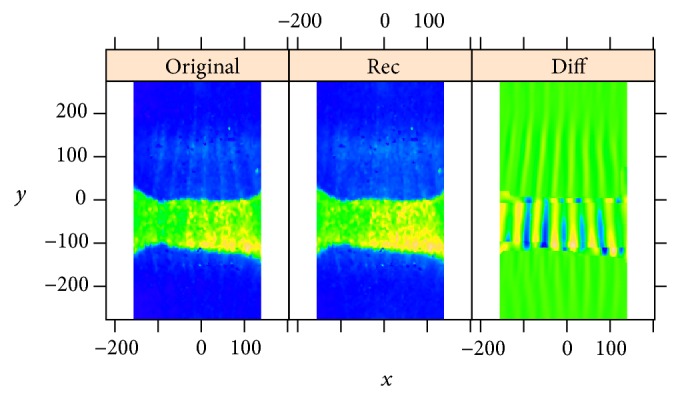
*sna*, combined image (both zones from Figures 14 and 15). ((a) to (d)): original image, reconstruction without stripes, and the difference. BDTNP embryo  v5-s10531-28fe05-07.pce.

A number of genes express in patterns which are more complex than the general AP variation seen with gap genes such as* hb* and* Kr*. To analyze crosstalk for such data, we introduce the shaped version of 2D-SSA. As an example,* snail* (*sna*) is expressed in a broad band along the ventral midline of the embryo (Figure 16, v5-s10531-28fe05-07, cy3_apical). Since* sna* shows a very sharp transition from expressing to nonexpressing regions, we analyzed these separately (Figure 14, expressing; Figure 15, nonexpressing). Analysis was conducted on a regular grid (step 0.5%), clipped 15% from left and right (as for Figure 9). For the central expressing zone (Figure 14), we used a window of 40 × 10; for the lateral nonexpressing zone (Figure 15), we used a window of 30 × 10.

**Figure 17:**
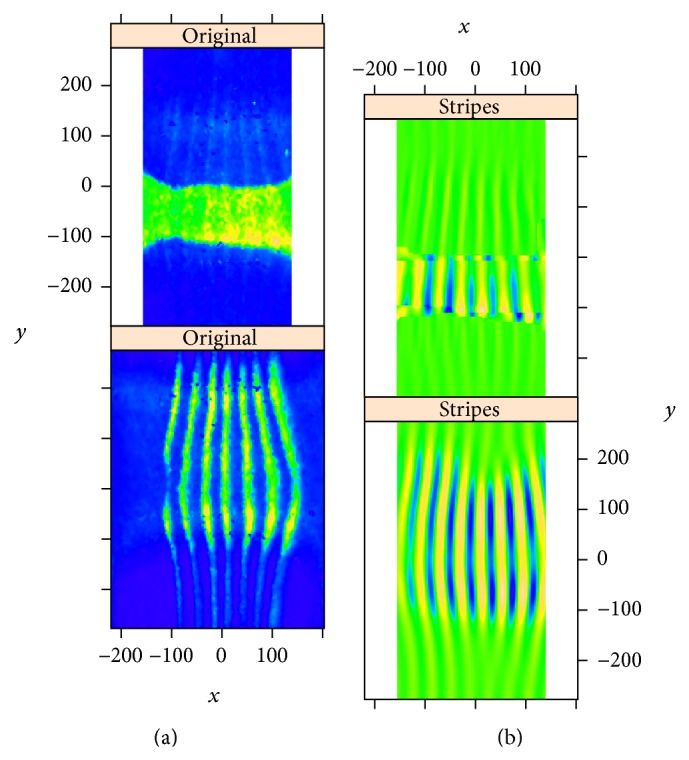
*sna* and* eve*: the original images (a) and the stripes (b),* sna* at top and* eve* at bottom.

Decomposition shows that the elementary components {3,4} (Figure 14) and {4,5, 16,17} (Figure 15) correspond to stripes, which come from the eve reference marker. Figures 14 and 15 show these stripe components and the effect of removing these stripes to reveal the* sna* signal. Figure 16 shows this for the complete* sna* image (combination of the expressing and nonexpressing zones). In this case, the stripe components from the* sna* image and from the* eve* marker image are in phase, indicating that this is a case of undercorrection in the unmixing algorithm (see Figure 17, where the original images and the stripe reconstructions are put together).

Thus, we have constructed a procedure for removing under- or overcorrections. Note that if an image does not contain stripes, images of elementary components also will not contain stripes and therefore can see if correction is necessary.

## 5. 3D Geometry of the Early Segmentation Pair-Rule Stripes

As discussed above, the early* Drosophila* embryo is roughly a prolate ellipsoid. Gene expression patterns defining the AP and dorsal-ventral (DV) axes are relatively independent. However, even clearly AP-varying patterns, such as the* eve* and* ftz* pair-rule striped patterns, display some degree of DV variation. This can be affected by deviations from ellipsoidal symmetry (e.g., embryos have a longer ventral surface (or “belly”) than dorsal surface) and also from variations in the axial ratio (see [4]).

Embryo-to-embryo variability in* eve* expression in the AP axis has been well documented and discussed in terms of the robustness of the developmental programme. However, such analysis has been in 1D. Analyzing 3D images, for example, with 2D-SSA, reveals new levels of variability.

**Figure 18:**
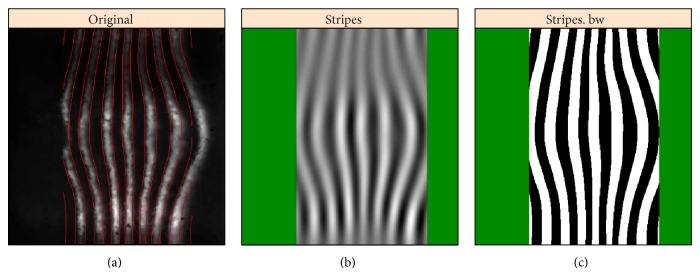
(a) original image, (b) reconstruction of strips, (c) conversion to black and white, according to positive or negative values on the intensity scale; black-white boundaries are shown as red lines on the original image. BDTNP embryo “v5_s10901-20ap06-11s10901.”

Figure 18 is an unfolded cylindrical projection of eve expression, showing the DV variation of the 7-stripe pattern, especially as the stripes bend around the ventral “belly” of the embryo (horizontal midline of image). To quantify the stripe geometry, we identify stripe boundaries at the threshold between positive and negative values on the intensity scale.

**Figure 19:**
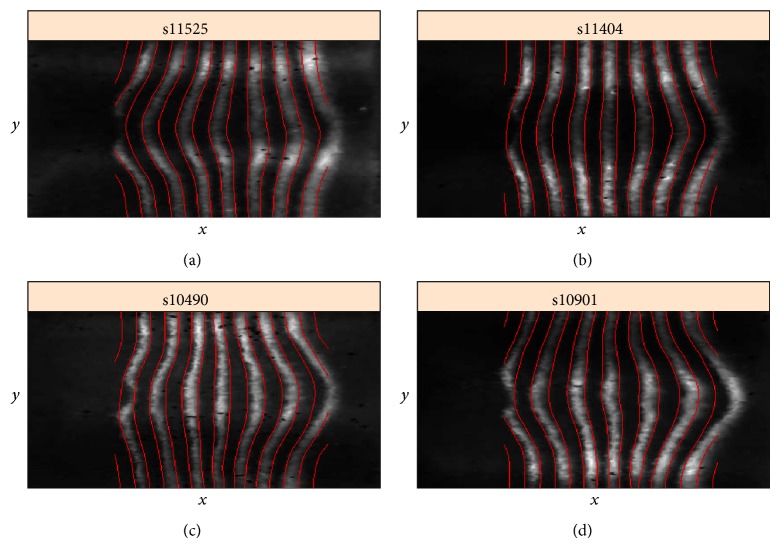
Four cases of the 3D geometry of eve expression stripes. Stripe 4 can be a forward “C”-shape (a), straight (b), a negative “C”-shape (c), or “S”-shaped (d). BDTNP embryo IDs are given on the images.

**Figure 20:**
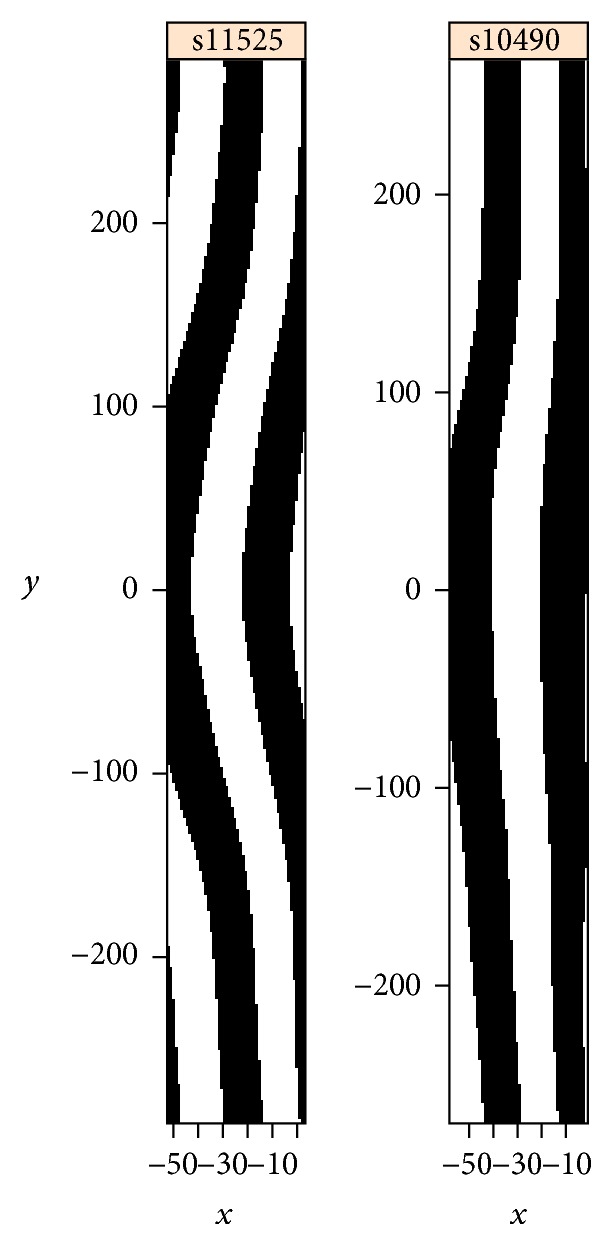
“C” and straight* eve* stripe 4 shapes, shown in black and white. BDTNP embryo IDs given on the images.

Using this boundary identification procedure, let us focus on the shape of the central (4th) eve stripe (Figure 19; the 4th stripe has minimal effect from the ellipsoidal to cylindrical projection). Preprocessing included interpolation of the cylindrical projection to a regular grid, clipping 25% of the image on the left and 15% on the right and using a 15 × 10 window. Applying circular 2D-SSA, we use components 2 and 3 to represent the striped expression. The 4th stripe is frequently straight across the ventral midline (Figure 19(b)) but can often show curvature as well. Curvature can be “C”-shaped, both forwards (Figure 19(a)) and backwards (Figure 19(c)), or “S”-shaped (Figure 19(d)). For clarity, Figure 20 shows the “C” and straight shapes in black and white and in the original aspect ratio.

Stripe 4 of* eve* is critical for subsequent segmentation events in fly development. These events need to be robust to the curvature variability reported here. It is currently unknown which mechanism might produce this robustness, but it warrants further investigation. For example, what is the correlation between the size of the ventral “belly” of the embryo and stripe 4 curvature? And does this suggest a “shape compensation” such that embryos can develop normally despite variable early geometry? (Systematic analysis should also be done to examine the possible contribution of experimental errors (e.g., fixation procedures) to stripe variability, which may involve comparison with live imaging techniques.)

## 6. Conclusions

This paper has shown the applicability of our new shaped and circular extensions of 2D-SSA to analyzing embryo images from a quantitative high-throughput project in developmental biology. We have shown that 2D-SSA can decompose images and classify components according to the gene of interest. This is an effective means for reducing the “crosstalk” between gene channels which arises in the imaging technique but can be amplified by the automated postprocessing unmixing algorithm.

Circular 2D-SSA is a critical extension for analyzing cylindrical data projections (accounting for periodic boundaries in “rectangular” images). Shaped 2D-SSA allows for the analysis of subregions of the image, important for analyzing complex expression patterns, complex geometries, and avoiding edge effects.

The procedure is performed under user control and can be adapted to an image's unique structure with a flexible choice of window shapes and sizes. This is currently a manual procedure and future work will focus on reliable automation of the process.

We have demonstrated that 2D-SSA can be used to extract signal and noise from images with both strong and weak over- or undercorrection of crosstalk. This is a significant tool for separating gene expression in multichannel images and for extracting residual noise for studying the stochastic aspects of gene expression. In particular, we have used SSA to separate low frequency genes of interest (the gap genes* hb* and* Kr*, and* sna*) from “bleed-through” crosstalk of the high-frequency pair-rule fiduciary markers (*eve* and* ftz*). In addition, we have shown how SSA components can be used to quantify* eve* stripes (in particular stripe 4) and how this reveals new types of variability in expression, leading to new insights into developmental mechanisms. These are all examples of how 2D-SSA can be applied—we expect them to be broadly generalizable to other cases of multichannel 3D data from* Drosophila* and other organisms.
